# Primary renal teratoma: a rare entity

**DOI:** 10.1186/1746-1596-8-107

**Published:** 2013-06-25

**Authors:** Karima Idrissi-Serhrouchni, Hinde El-Fatemi, Aziz El madi, Khadija Benhayoun, Laila Chbani, Taoufik Harmouch, Youssef Bouabdellah, Afaf Amarti

**Affiliations:** 1 Department of Pathology, Hassan II University Hospital, Fez 30000, Morocco; 2 Department of Pediatric Surgery, Hassan II University Hospital, Fez 30000, Morocco

**Keywords:** Teratoma, Immature, Wilms, Kidney, Extragonadal

## Abstract

**Abstract:**

Teratomas are neoplasms that arise from pluripotent cells and can differentiate along one or more embryonic germ lines. Renal teratoma is an exceedingly rare condition. Teratomas commonly arise in the gonads, sacrococcygeal region, pineal gland, and retroperitoneum. They present mainly as an abdominal mass with few other symptoms. Majority of the tumors are benign, situated on the left side and para renal, occasional lesions are bilateral. If diagnosed early, they are amenable to curative excision.

Renal teratomas are rare and most have been dismissed as cases of teratoid nephroblastomas or retroperitoneal teratomas secondarily invading the kidney. The differentiation between these two neoplasms in the kidney is often problematic.

We present a case of intrarenal immature teratoma in a six-month-old baby girl.

**Virtual slides:**

The virtual slides for this article can be found here: http://www.diagnosticpathology.diagnomx.eu/vs/1746249869599954.

## Background

Teratomas are neoplasms that arise from pluripotent cells and can differentiate along one or more embryonic germ lines [1]. Renal teratoma is an exceedingly rare condition [1]. Teratomas commonly arise in the gonads, sacrococcygeal region, pineal gland, and retroperitoneum. The proximity of the genital ridge to the nephrogenic anlage may partly explain how germ cells could be displaced within the kidney [2].

To the best of our knowledge, our patient is the second known case with an immature teratoma arising from ectopic kidney the first one was developed in a horseshoe kidney.

We present a case of intrarenal immature teratoma in a six-month-old baby girl and discuss the pathology of this rare entity.

## Case report

A six-month-old baby girl was admitted in the department of paediatric surgery of Hassan II university hospital in Morocco, with the complaints of abdominal distension and pain since one month. On examination her weight was 5.5 kgs and her abdomen was hugely distended. On palpation, a firm mass occupying all of the left lumb of the abdomen was palpable, which was tender, moving with respiration and the margins were irregular. Both the renal and liver functions as well as the findings of hematological studies were within the normal limits. A chest x-ray showed no abnormality. Abdominal ultrasonography demonstrated a pelvic left kidney measuring 18 cms in diameter with an important expansion of the excretory cavities and internal cystic and solid changes. Computed tomography showed a mass containing low-density areas of the left kidney. The right kidney was normal. The patient did not found any more tumors in the other organs. Because the most frequent tumor of the kidney occurring in newborns is congenital mesoblastic nephroma and patients with ectopic kidneys have a high risk for Wilms’ tumor, a diagnosis of either mesoblastic nephroma or Wilms’ tumor arising from ectopic kidney was tentatively made, and she was thus considered to be indicated for surgery.

Patient was operated upon; laparotomy was done through a median underumblical incision. A large partly cystic and partly solid mass with extensive areas of haemorrhage in the cystic areas was present in the left pelvic region. It was roughly spherical in shape. Postoperative recovery of the patient was uneventful and she was discharged on 10th postoperative day. The specimen was sent for histopathological examination.

**Figure 1:**
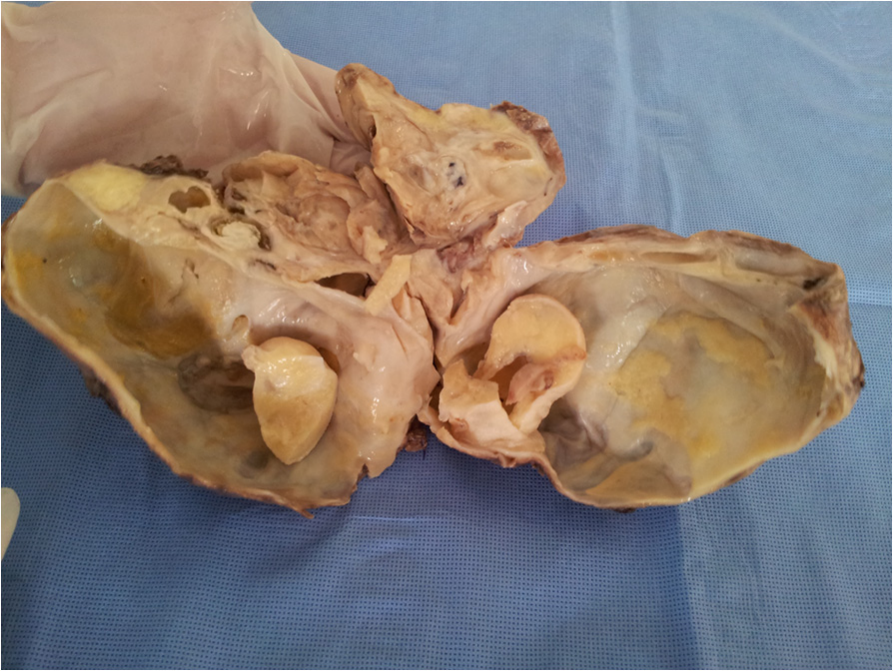
**Gross view of lesion shows an encapsulated tumor (18 cm) with a yellowish tan cut surface.** The interior was cystic with solid structures.

Grossly a large mass was received measuring 18×12×8 cms in maximum dimensions. It weighed 200 Gms, linked to an ureter of 3×0,2 cms. On sectioning a multilocular circumscribed lesion was identified measuring 2,5 at 10 cms. It was attached to the cystic wall, which focally showed thickened brownish areas in the wall (Figure 1). The entire kidney was replaced by the lesion, only a thin rim of renal parenchyma was identified at the periphery. Multiple sections were taken from the cyst wall and from the nodular lesion and vessels.

**Figure 2:**
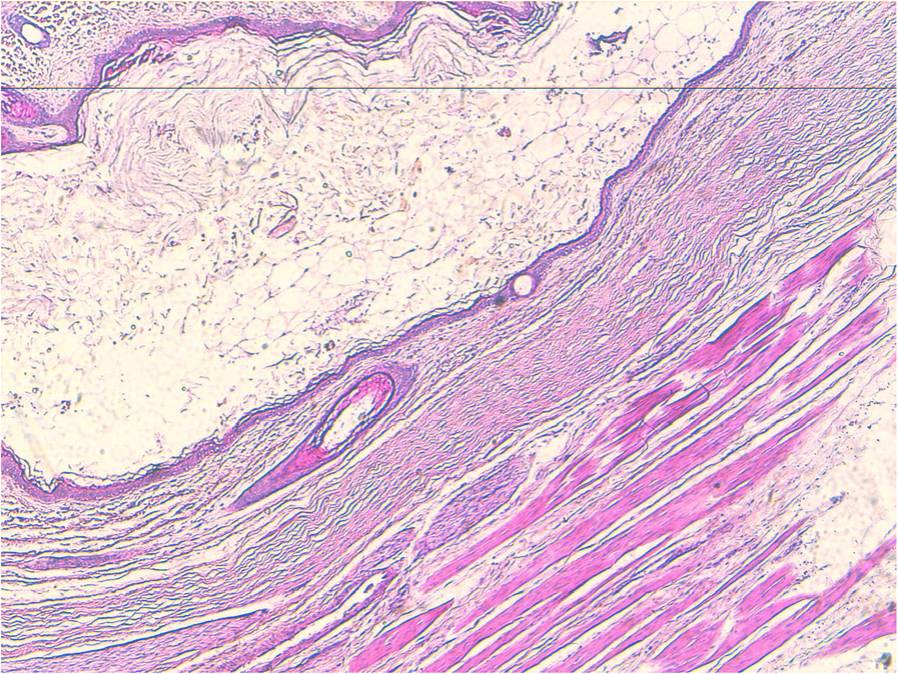
**Teratomatous components of keratinizing stratified squamous epithelium with skin adnexae.** Original magnification ×100 (H&E).

**Figure 3:**
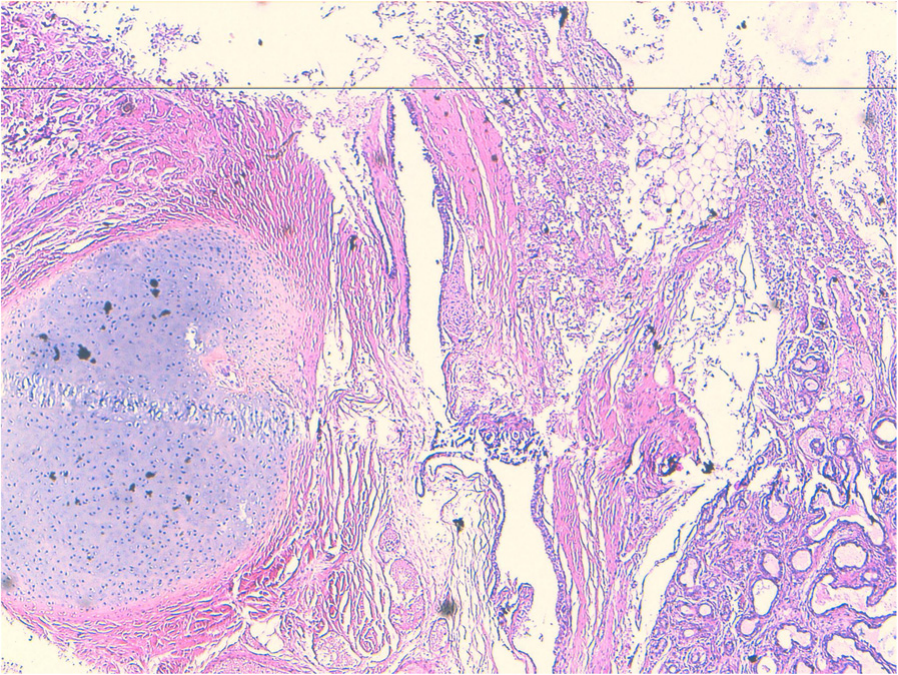
**Teratomatous components of cartilage and mucinous columnar epithelium.** Original magnification ×100 (H&E).

**Figure 4:**
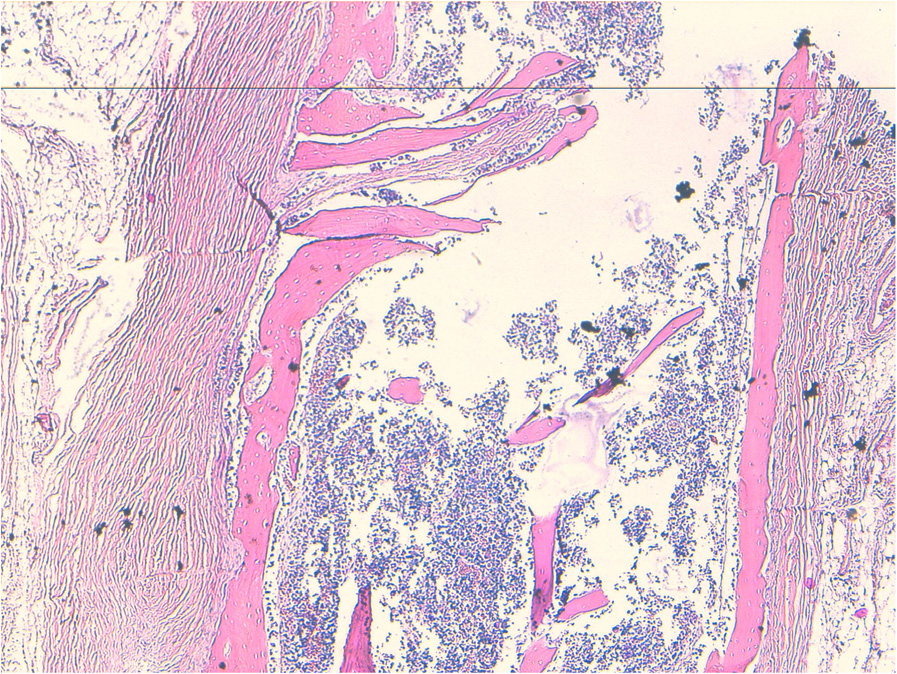
**Teratomatous components of bone.** Original magnification ×100 (H&E).

**Figure 5:**
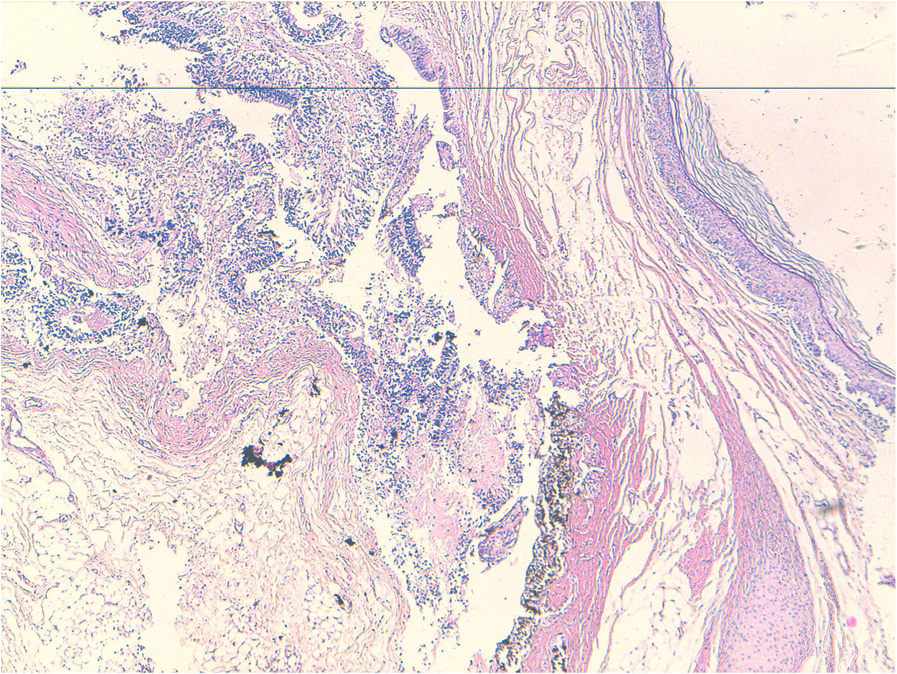
Teratomatous components of Melanin, neuroglial cells and immature neuroectodermal tissue Original magnification ×100 (H&E).

**Figure 6:**
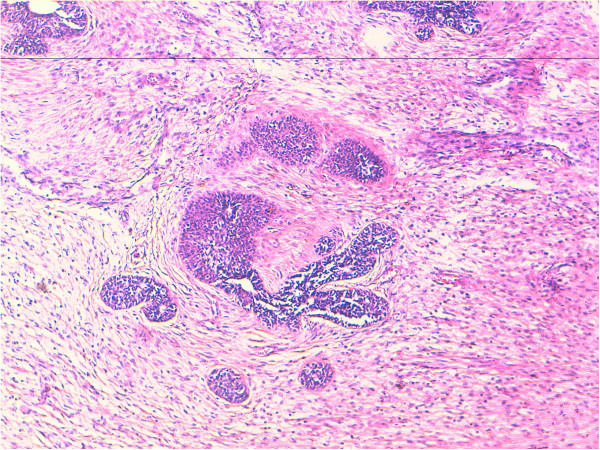
**Teratomatous components of neuroglial cells and immature neuroectodermal tissue rosette-like.** Original magnification ×100 (H&E).

Microscopically small cystic spaces lined by keratinizing stratified squamous epithelium with skin adnexae were identified (Figure 2). The solid areas showed large foci of cartilage, mucinous columnar epithelium (Figure 3) and bone formation (Figure 4). Melanin containing cells and neuroglial cells with occasional foci of immature neuroectodermal tissue were also identified (Figures 5 and 6). The cyst wall was thick fibromuscular without any lining. Sections from solid areas in the cyst wall revealed immature renal tissue. The diagnosis of immature teratoma was retained.

## Discussion

Teratomas are rare neoplasms (incidence 0.7/100.000 children/year) with tissue derivatives of all three germ layers [3]. Teratomas mostly occur in the ovaries, the sacrococcygeal region, the testes, the central nerval system, the mediastinum, and only rarely in other locations with less than 5% occurring in the abdomen [2,3]. Kidney is one of the least common locations for teratomas and other germ cell tumors [1]. Teratomas are thought to have been present since birth, or even before birth, and are therefore considered as congenital tumors [3]. Retroperitoneal teratomas exhibit a bimodal presentation, with peaks in the first six months of life and early adulthood [2]. Literature does not reveal a side or gender predilection and almost equal incidence in males and females have been reported [4]. The first reported case of teratoma of kidney was in 1934, when Mc Curdy described this entity in a seven-week-old child with Prune-Belly syndrome [5]. Since, fifteen prior case reports were found in a MEDLINE search and we additioned our case to this data, the main findings for these cases are summarized in Table 1[6-20].

**Table 1:** Clinical-characteristics, radiographic and pathologic features of primary teratoma of the kidney

**Source, year**	**Side**	**Age**	**Sex**	**Clinical presentation**	**Radiographic features of renal mass**	**Components of teratoma**
Kojiro et al., [6]	Left kidney	40 years	Male	Epigastric pain, nausea, no carcinoid syndrome	IVU: Marked dilatation of left renal pelvis	Mucous secretory glands, columnar epithelium, mature hyaline cartilage, smooth muscle
Fetissof et al., [7]	Right kidney	65 years	Male	Fever, no carcinoid syndrome	IVU: Displaced left kidney and non-visualized right kidney	Transitional and mucinous columnar epithelium, smooth muscle, ossified chondroid plaques, nerve bundles with ganglion cells
Lodding et al., [8]	Right kidney	23 years	Male	Abdominal pain, no carcinoid syndrome	CT: Calcification in horseshoe kidney	Mature bone
LIU et al., [5]	Left kidney	2 years	Female	Poor appetite and poor activity 1 week in duration	CT: Huge tumor in the left kidney with calcification and necrosis	Yolk sac tumor and immature teratoma
Singer et a.l, [9]	Left kidney	2 months	Male	Constipation and a palpable left flank mass	CT : Heterogeneous upper pole left renal neoplasm	Mature teratoma with rare
						foci of immature elements
Govender et al., [10]	Right kidney	3 years	Female	Bilateral coarse crackles and a wheeze. Abdominal distension	CT: Large tumour involving The right side of the abdomen	Mature renal teratoma and a synchronous malignant neuroepithelial tumour of the ipsilateral adrenal gland
Otani et al., 2001 [11]	Left kidney	6 years	Male	Mass of a left side abdominal	CT: Multiple cystic masses	Keratinizing squamous epithelium with hair follicles, shafts and sebaceous glands. atrophy of the adjacent renal parenchyma, with partially dysplastic and angiomyolipoma
Yoo et al., [12]	Left kidney	30 years	Female	Abdominal pain, no carcinoid syndrome	CT: Dense calcification with minimal contrast enhancement	Mucinous columnar epithelium, smooth muscle, mature bone
Yaqoob et al., [13]	Left kidney	2 months	Female	Firm mass on left side of the abdomen, which was crossing to the right side as well	X-ray : soft tissue mass with areas of calcification Ultrasound : complex mass partly cystic and partly solid	Cystic spaces lined by keratinizing stratified squamous epithelium with skin adnexae
Kim et al., [14]	Right kidney	39 years	Female	Asymptomatic, incidental renal mass, no carcinoid syndrome	CT: mass relatively well demarcated and incompletely marginated by a thin hypodense rim with globular calcifications	Mucinous columnar and Pseudostratified columnar epithelium with occasional cilia, mature bone
Kurzer et al., [15]	Right kidney	58 years	Female	Asymptomatic, incidental renal mass, no carcinoid syndrome	CT: Round mass, smooth, and well marginated, with two solid clumps of calcifications	Transitional, colonic, squamous, and nonspecific cuboidal epithelium, mature adipose, focal osseous metaplasia
Choi et al., [16]	Right kidney	4 years	Female	Right-sided abdominal mass	US : tumor with intermediate echogenicity and an ovoid, hypoechoic central region	Adipose tissue. squamous epithelial with abundant keratinous flakes. pilosebaceous adnexal elements
Ledo et al., [17]	Left kidney	4 months	Male	Left -sided abdominal mass	CT: Heterogeneous tumor, amorphous calcifications	Mature teratoma
Kyoko et al., [18]	Right kidney	6 days	Female	Hard mass of 6 × 6 cm in diameter, in the right upper abdomen	CT: mass containing low-density areas from the isthmus to the right lower pole of a horseshoe kidney	Mature tissu contained neuroepithelial components
Henry B Armah et al., [19]	Right kidney	35 years	Female	Right flank pain, right costovertebral angle tenderness, no carcinoid syndrome	CT: Exophytic, round, well-circumscribed mildly complex hypodense with globular calcifications	Urothelial-type and colonic epithelium,focal mature bone
Madhumita et al., [20]	Right kidney	1 month	Male	Mass in the right side of theabdomen since birth	CT: soft tissue density mass with fat and fluid components arising from the right kidney. Multiple calcifications duplication of the cecum and Appendix	Mature benign teratoma
Current case	Left kidney	6 months	Female	Abdominal distension and pain	US: pelvic left kidney measuring 18 cms in diameter with an important expansion of the excretory cavities and internal cystic and solid changes CT: mass containing low-density areas of the left kidney	keratinizing stratified squamous epithelium with skin adnexae, cartilage, mucinous columnar epithelium,bone, melanin containing cells and neuroglial cells with occasional foci of immature neuroectodermal tissue

*IVU* IntraVenous Urography, *CT* Computed tomography, *US* ultrasonography.

The clinical symptoms are an abdominal mass, abdominal pain, abdominal discomfort, pyelonephritis, and constipation [18]. Occasionally, the tumor is present antenatally and diagnosed at birth, these neonatal teratomas have a higher incidence of malignancy than those in older children [4,21]. The diagnostic algorithm was palpation of a solid flank mass, in our case it was a pelvic mass, plain X-ray to demonstrate calcification or formed bony components like teeth and phalanges which are pathognomonic. Ultrasound was sufficient to define the relationships of the tumor for planning surgery. CT scan was used to define the extent of the disease in lesions occupying both sides of the retroperitoneum and those tumors where calcification is not seen on plain X-ray. Hayasaka and Yamada have reported internal homogeneity, fat density, cyst formation and calcification to be important predictors of a benign retroperitoneal tumor on CT [4]. The role of magnetic resonance imaging in such tumors is unclear. The single reported case in which magnetic resonance imaging was performed noted a heterogeneous mass with low signal intensity [20]. Papanicolau and Yoder advocate angiography, inferior venacavography and needle biopsy for the accurate diagnosis of these tumors. Serum alpha-fetoprotein formed a useful marker of monitoring recurrence [4].

In mature teratomas skin with dermal appendages, bronchial structures with bronchial glands and cartilage, neuroglial tissue, and teeth are commonly present and regarded as evidence of organogenesis. In the other hand immature teratoma contained neuroepithelial components with an embryonic appearance and ependymal rosette-like [10].

Only six cases of primary carcinoid tumor arising in a mature teratoma of the kidney have been reported in the world medical literature to date [19]. Otani et al. reported a case of a six-year-old boy with intrarenal cystic teratoma, associated with renal dysplasia [1,11]. M. Mukhopadhyay et al. reported a renal teratoma with duplication of the cecum and appendix. Various congenital anomalies have been reported with renal teratoma [16,20]. Developmental anomalies increase the risk of teratoma [16,20].

The differential diagnosis of intrarenal teratoma include Wilms’ tumor [22,23]. The both of these tumors originate from the mesodermal metanephrogenic blastema, and in histological examinations they are similar. Wilms’ tumor can contain a variety of heterologous elements with histologic findings of blastemal, stromal, and epithelial cell types [10,18]. Therefore, a differential diagnosis between intrarenal teratoma and teratoid nephroblastoma is difficult even when making a pathological diagnosis because it can only be made based on a detailed analysis of the tumor after resection. It is therefore highly possible that anticancer drug treatment is required for Wilms’ tumor [18,20].

The second differential diagnosis is congenital mesoblastic nephroma [24]. Sonographically, classic congenital mesoblastic nephroma may appear as a hypoechogenic tumor with an echogenic rim, but it sometimes may also appear as a heterogenous solid mass like teratoma. A cut section of congenital mesoblastic nephroma reveals an unencapsulated mass having a whorled pattern. As a result, a differential diagnosis between intrarenal teratoma and congenital mesoblastic nephroma may thus be found to be macroscopically possible. Moreover, the histologic features of congenital mesoblastic nephroma mainly consist of elongated spindleshaped cells arranged in interweaving bundles with renal glomeruli and tubules. Classical congenital mesoblastic nephroma has an excellent prognosis in patients younger than the age of 3 months. A radical resection of the tumor is the therapy of choice, and it is usually curative.

Other differential diagnosis is cystic neuroblastomas [25]. This neoplasm is characterized by its cystic appearance with no calcification inside and just a small portion of solid tissue [26].

An immature teratoma has a strong resemblance to small, blue round cell tumors which commonly include Wilm’s tumor, metanephric adenoma, lymphoma, peripheral neuroectodermal tumor and rhabdomyosarcoma; and rarely metastatic small cell tumors from lung [23]. It may be present as primary renal cell sarcoma and poorly differentiated renal carcinoma [27].

Beckwith suggests that for a tumor to be termed a renal teratoma it should meet two criteria: (a) the primary tumor should be unequivocally of intrarenal origin, the entire lesion should be contained within the renal capsule and there should be no teratomas in remote sites which might have metastasized to the kidney. (b) the tumor should exhibit unequivocal heterotopic organogenesis [13]. Our case report fulfils both these criteria.

An accurate histologic diagnosis is very important. Complete excision of the tumor mass is recommended and anticancer drug treatment is unnecessary. Patients with pure immature teratomas can be effectively treated with a surgical excision alone because the 3-year event-free survival is more than 85% [18].

Follow-up data after surgical removal for intrarenal teratomas in children are limited. Mature teratomas are usually benign, but they have the potential for malignant transformation. All patients with the diagnosis of benign teratoma should undergo regular long-term follow-up examinations [17].

## Conclusion

The purpose of this review was to stress on the fact that though primary renal teratomas are extremely rare, this entity must be taken into consideration in the differential diagnosis of any renal mass in childhood.

### Consent from the patient

Written informed consent was obtained from patient’s parents for publication of this case report.

## Abbreviations

CT: Computed tomography.

## Competing interests

The authors declare that they have no competing interests.

## Authors’ contributions

All authors read and approved the final manuscript.
